# Recombinant Human Lysozyme Attenuates Hyperosmolarity-Induced Dry Eye Disease by Suppressing Oxidative Stress and Modulating NLRP3 Inflammasome Signaling

**DOI:** 10.3390/ijms27209297

**Published:** 2026-10-20

**Authors:** Yuqing Jiang, Weili Hu, Lingxiao Yi, Minal Thacker, Qiyun Wu, Yuan Li, En Gao, Jingfang Bian, Chi Wai Do, Thomas Chuen Lam, Man Sau Wong, Liping Zhou

**Affiliations:** 1 Centre for Eye and Vision Research (CEVR), 17W Hong Kong, Science Park, Hong Kong; 2 PolyU–HuiKang Joint Laboratory, The Hong Kong Polytechnic University, Kowloon, Hong Kong; 3 School of Optometry, The Hong Kong Polytechnic University, Kowloon, Hong Kong; 4 Department of Food Science and Nutrition, The Hong Kong Polytechnic University, Kowloon, Hong Kong; 5 Research Centre for Chinese Medicine Innovation, The Hong Kong Polytechnic University, Kowloon, Hong Kong

**Keywords:** dry eye disease, lysozyme, anti-inflammation, NLRP3 inflammasome, anti-oxidation

## Abstract

Given the escalating global prevalence of dry eye disease (DED) and the inadequacy of current palliative management, this study was to evaluate thetherapeutic potential of recombinant lysozyme (rlysozyme) and investigate underlying mechanisms. Human corneal epithelial cells (HCEC) were subjected to hyperosmotic stress and treated with graded rlysozyme. Cell viability, wound healing, barrier integrity, oxidative stress, cytokines, and mitochondrial function were evaluated by MTS and scratch assays, immunofluorescent staining, ELISA, qRT-PCR, and flow cytometry. Results: No cytotoxicity was observed at any of the tested concentrations (2.76 × 10^−9^–2.76 × 10^−4^ M) in HCEC. In hyperosmolarity-induced DED cells, rlysozyme at the highest concentrations (2.76 × 10^−4^ M) significantly increased cell viability by 12.30% (*p* < 0.001) and promoted corneal repair by 45.04% (*p* < 0.05), which were comparable to those of 0.4% hyaluronic acid (the positive control). It also improved via restoring ZO-1 organization. Mechanistically, qRT-PCR results revealed that rlysozyme reduced mRNA expression of IL-1β (by 28.7%), TNF (by 39.2%) and key NLRP3 inflammasome components, including NLRP3 (by 62.0%) and PYD and CARD domain-containing protein gene(*PYCARD*, 21.4%), while caspase-1 (*CASP1*) and IL-6 expression remained unchanged. Moreover, oxidative stress was attenuated, as evidenced by significant decreases in reactive oxygen species (ROS, by 59.1%), 8-hydroxy-2-deoxyguanosine levels (8-OHdG, by 23.6%) and total matrix metalloproteinase-9 (MMP-9, by 64.3%) protein levels (*p* < 0.001), while mitochondrial membrane potential was assessed qualitatively. In conclusion, this study demonstrates thatrlysozyme effectively enhanced corneal epithelial cell viability, promoted wound healing, and restored ZO-1 structural organization without inducing cytotoxicity. These protective effects were associated with downregulation of NLRP3 inflammasome-related genes and attenuation of oxidative stress, supporting further investigation of its therapeutic potential in DED.

## 1. Introduction

Dry eye disease (DED) is a prevalent multifactorial ocular disorder characterized by a range of symptoms such as ocular surface dryness, irritation, blurred vision, and inflammation that considerably impact daily life [1,2]. Epidemiological studies estimate the prevalence of DED at up to 50% worldwide [3] and an even higher rate has increasingly been reported in younger populations, largely driven by the ubiquitous adoption of digital devices, contact lenses, and concomitant lifestyle changes [4]. Despite its prevalence, current clinical management remains largely palliative, focusing on symptom relief rather than addressing the underlying pathophysiology. Consequently, there is a critical unmet need for targeted therapeutic strategies that can effectively restore ocular surface homeostasis and provide long-term relief.

Impairment of tear film stability is a hallmark of DED pathophysiology [5]. Behavioral and environmental factors drive this destabilization through distinct mechanisms. Prolonged digital screen use reduces spontaneous blinking, while contact lens wear disrupts the protective tear lipid layer. Together with dry ambient environments, these disruptions compromise tear film stability, accelerating tear evaporation and elevating tear osmolarity [6]. The resulting hyperosmolar environment directly stimulates corneal and conjunctival epithelial cells, inducing oxidative stress and increasing intracellular reactive oxygen species (ROS) levels [7]. Excessively produced ROS not only directly damage the ocular surface epithelial cells but also act as key signaling molecules to activate the NOD-, LRR-, and pyrin domain-containing protein 3 (NLRP3) inflammasome [8], which in turn triggers caspase-1-dependent maturation of interleukin-1 β (IL-1β), upregulation of pro-inflammatory cytokines such as tumor necrosis factor α (TNF-α) and interleukin-6 (IL-6), and mitochondrial dysfunction [9]. This self-perpetuating “vicious cycle” of oxidative stress and NLRP3-mediated inflammation underscores the necessity of a multifunctional therapeutic agent.

Lysozyme is a 14 kDa cationic antimicrobial enzyme that is abundantly present in human tears, where it serves as a crucial component of the innate immune defense of the ocular surface [10]. Reduced lysozyme levels have been consistently observed in the tears of patients with both aqueous deficient and evaporative DED [10,11,12]. This deficiency is believed to compromise the ocular surface’s defense, further destabilize the tear film, and exacerbate inflammatory cascades [11]. A study of human retinal pigment epithelial ARPE19 cells demonstrated that the CRISPR/Cas9-mediated deletion of lysozyme significantly impaired its antimicrobial activity, which was accompanied by an upregulation of TNF-α pathway-mediated IL-6 expression [13]. Similarly, lysozyme preconditioning in human corneal epithelial cells (HCEC) under a model mimicking SARS-CoV-2 infection almost completely inhibited the inflammatory cascade driven by the upregulated TNF-α/IL-6 pathway [14]. Additionally, lysozyme exhibits direct antioxidative properties by acting as a “sacrificial antioxidant” [15]. In vivo evidence from transgenic zebrafish models further confirms its protective role against oxidative stress through suppression of ROS generation and modulation of oxidative stress response genes [16].

Collectively, these properties suggest that restoring lysozyme could mitigate oxidative damage and NLRP3 inflammasome activation, offering a promising therapeutic approach for DED. This study aims to investigate the therapeutic potential of lysozyme in DED and address the underlying mechanisms by using an *in vitro* model.

## 2. Results

### 2.1. Rlysozyme Is Non-Cytotoxic and Significantly Rescues Hyperosmolarity-Induced Cell Death

**Figure 1:**
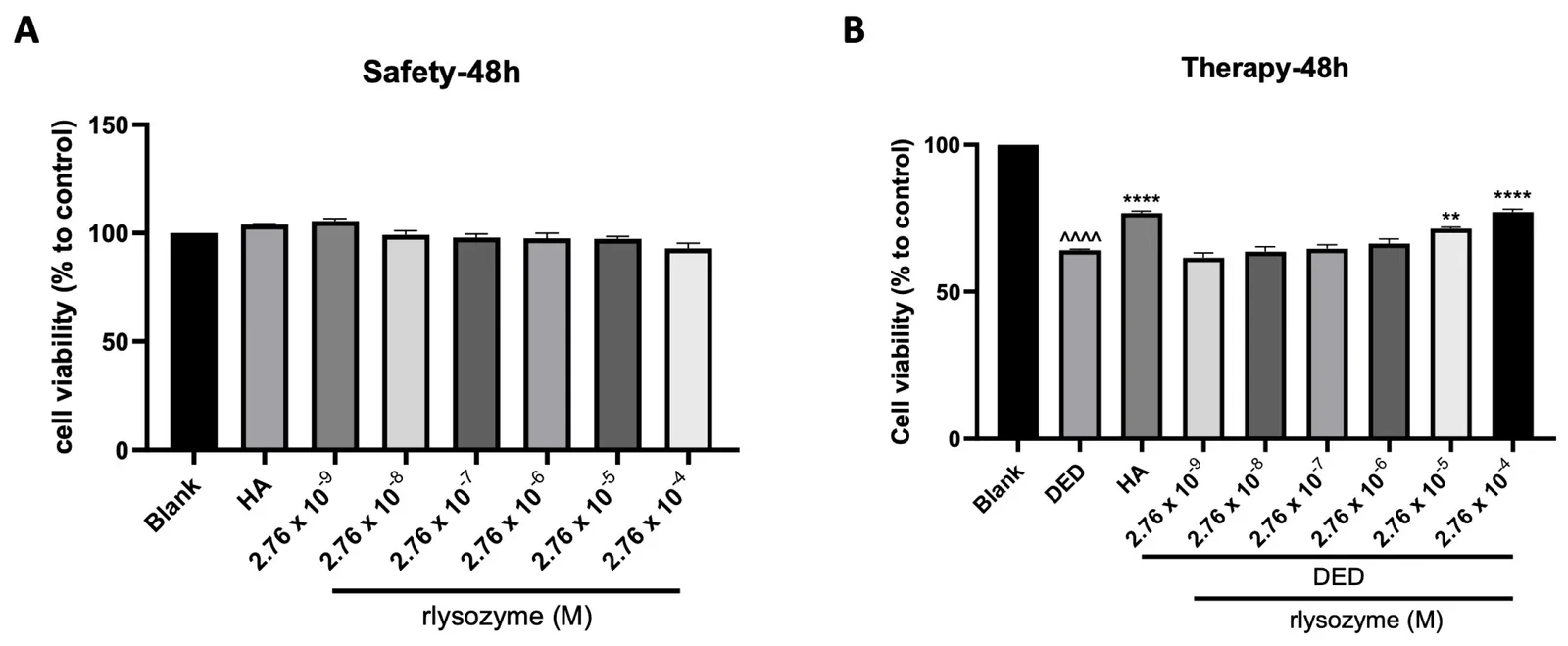
rlysozyme showed no detectable cytotoxicity under the tested conditions and reduced hyperosmolarity-induced cell death at higher concentrations. (**A**) Different concentrations of rlysozyme did not show significant difference in cell viability to blank group, showing no toxicity in HCEC. (**B**) Hyperosmotic-induced DED showed significant difference (^^^^^^ *p* < 0.0001 vs. blank group) in cell viability while rlysozyme with higher concentrations showed therapeutic function for DED (**** *p* < 0.0001, ** *p* < 0.01 vs. DED group). *N* = 4; inter-group difference was determined by one-way ANOVA with Tukey’s post hoc test for multiple comparisons and error bars denote SEM.

Throughout this study, the blank group refers to cells maintained in isosmotic medium (312 mOsm) without any treatment, the DED group denotes cells exposed to a hyperosmotic medium (450 mOsm) without rlysozyme, and 0.4% hyaluronic acid (HA) served as the positive control. As shown in Figure 1A, no significant differences in cell viability were observed at any of the tested concentrations of rlysozyme compared with the blank group, indicating no detectable cytotoxicity under the tested conditions. Under hyperosmotic stress, the cell viability of HCEC was significantly reduced by more than 35% compared to the blank group (Figure 1B, *p* < 0.001 vs. blank), but was significantly restored by rlysozyme at higher concentrations (2.76 × 10^−5^ M and 2.76 × 10^−4^ M) (Figure 1B, *p* < 0.01 and *p* < 0.0001 vs. DED, respectively), reaching levels comparable to those of hyaluronic acid (HA, positive control) (Figure 1B, *N* = 4).

### 2.2. Rlysozyme Significantly Promotes Wound Healing and Maintains Tight Junction Protein Expression

**Figure 2:**
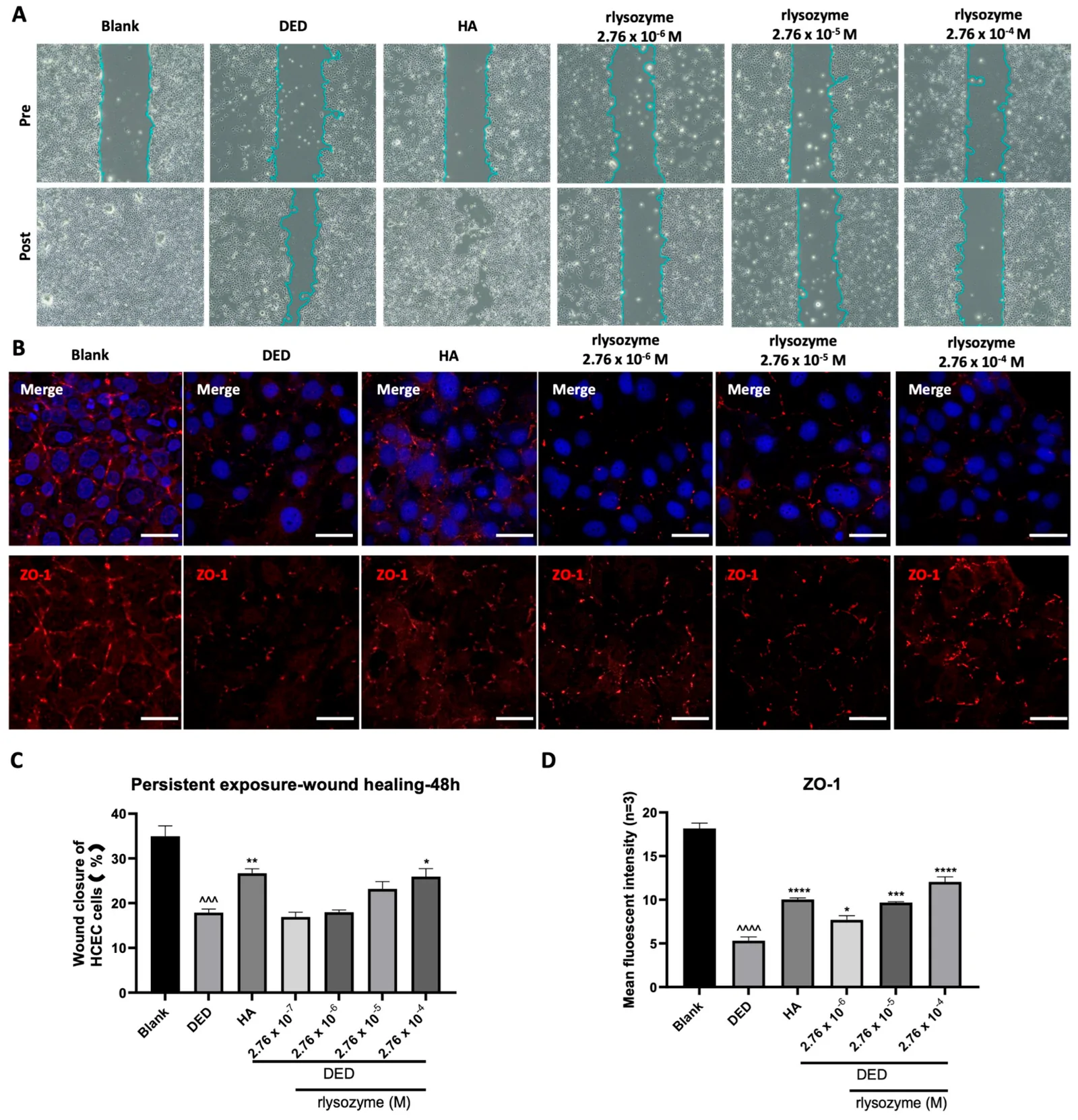
rlysozyme significantly promotes wound healing and tight junction. The wound healing capacity and tight junction of HCEC was assessed under hyperosmotic conditions simulating DED. (**A**) Representative phase-contrast images of the scratch-wound healing assay in HCEC. The dashed teal lines demarcate the wound margins at 0 h (Pre) and 48 h (Post) after treatment with blank, DED, HA, and varying concentrations of rlysozyme (2.76 × 10^−6^ M to 2.76 × 10^−4^ M). (**B**) Immunofluorescence staining of the tight junction protein ZO-1 (red) in HCEC across different treatment groups. Nuclei were counterstained with DAPI (blue). Scale bar 10 µm. (**C**) A significant difference in wound closure % was observed between the HA treatment group and the rlysozyme treatment group with the highest concentration (2.76 × 10^−4^ M). *N* = 4; inter-group difference was determined by one-way ANOVA with Tukey’s post hoc test for multiple comparisons and error bars denote SEM. (**D**) Bar chart shows quantification of ZO-1 expression level in HCEC. The DED group exhibited a significant decrease in ZO-1 expression compared to the blank group (^^^^^^ *p* < 0.0001). Treatment with HA significantly increased ZO-1 expression (**** *p* < 0.0001 vs. DED). rlysozyme treatment at concentrations of 2.76 × 10^−6^ M and 2.76 × 10^−5^ M also led to a significant increase in ZO-1 expression compared to the DED group (* *p* < 0.05 for 2.76 × 10^−6^ M; *** *p* < 0.001 for 2.76 × 10^−5^ M). The highest concentration tested, 2.76 × 10^−4^ M, showed a significant increase in ZO-1 expression (**** *p* < 0.0001 vs. DED). *N* = 3; inter-group difference was determined by one-way ANOVA with Tukey’s post hoc test for multiple comparisons and error bars denote SEM. Statistical significance is indicated as follows: ^^^ *p* < 0.001 and ^^^^ *p* < 0.0001 vs. the blank group; * *p* < 0.05, ** *p* < 0.01, *** *p* < 0.001, and **** *p* < 0.0001 vs. the DED group.

Since DED involves disruptions in ocular surface integrity and causes corneal barrier dysfunction, we next assessed whether rlysozyme could promote corneal repair and restore tight junction organization. As shown in Figure 2, rlysozyme at the highest concentration (2.76 × 10^−4^ M) significantly accelerated epithelial wound closure in hyperosmolarity-induced DED in HCEC, achieving 74.23% wound closure (Figure 2C, *p* < 0.05 vs. DED), compared to 51.18% in the untreated DED group (Figure 2C, ***p < 0.001 vs. blank, N = 4).

Tight junction protein Zonula Occludens-1 (ZO-1) is essential for maintaining corneal epithelial barrier and its disruption is a hallmark of impaired wound healing in DED. Therefore, we next examined the effect of rlysozyme on ZO-1 organization. As shown in Figure 2B, ZO-1 staining was continuous and intact in the blank group, whereas it was fragmented and discontinuous in the DED group. Treatment with rlysozyme (2.76 × 10^−6^ to 2.76 × 10^−4^ M) markedly restored the continuous and intact pattern of ZO-1 staining. Quantitative analysis (Figure 2D) further confirmed a significantly higher fluorescence intensity of ZO-1 in the rlysozyme group than that in the DED group (*p* < 0.05 vs. DED), indicating that rlysozyme effectively restored ZO-1 protein expression and structural continuity in the hyperosmolarity-induced DED.

### 2.3. Rlysozyme Downregulates NLRP3 and IL-1β Expression

**Figure 3:**
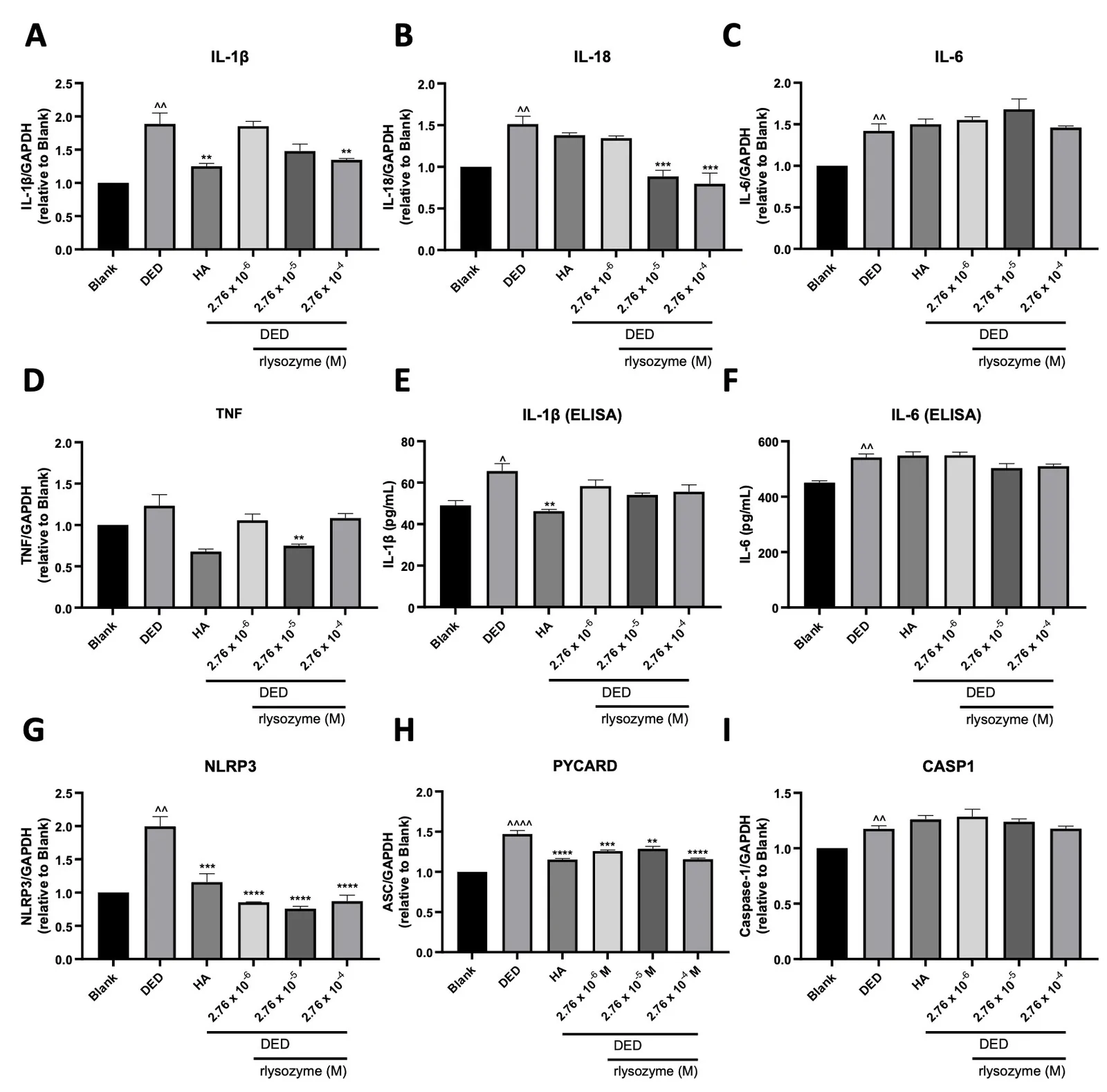
rlysozyme downregulates the NLRP3 inflammasome-related mRNA expression. qPCR results demonstrate the relative mRNA expression levels of *IL-1β* (**A**), *IL-18* (**B**), *IL-6* (**C**), *TNF* (**D**), *NLRP3* (**G**), *PYCARD* (**H**) and *CASP1* (**I**) in HCEC treated with different concentrations of rlysozyme. Each bar represents the mean expression level normalized to the housekeeping gene (*GAPDH*), with error bars indicating the SEM from three independent experiments. Significant differences were observed in the mRNA expression of *NLRP3* (**** *p *< 0.0001), *PYCARD* (**** *p *< 0.0001), *IL-1β* (** *p *< 0.01), *TNF* (** *p *< 0.01) compared to the DED group. (**C**,**I**) No significant differences in the mRNA expression of *IL-6* and *CASP1* were observed among the treatment groups. ELISA results showing the protein levels of IL-1β (**E**) and IL-6 (**F**) in pg/mL. No significant differences were observed in the protein levels of IL-1β and IL-6 compared to the DED group in rlysozyme treatment group. *N* = 3; inter-group difference was determined by one-way ANOVA with Tukey’s post hoc test for multiple comparisons. Statistical significance is indicated as follows: ^ *p* < 0.05, ^^ *p* < 0.01, and ^^^^ *p* < 0.0001 vs. the blank group; ** *p* < 0.01, *** *p* < 0.001, and **** *p* < 0.0001 vs. the DED group.

As shown in Figure 3, hyperosmolar stress significantly upregulated the mRNA expression of pro-inflammatory cytokines in HCEC. In particular, IL-1β, interleukin-18 (IL-18) and IL-6 mRNA levels were markedly elevated in the DED group compared with the blank group (Figure 3A–C, *p* < 0.05 vs. blank), while the increase in TNF mRNA did not reach statistical significance (Figure 3D). At the protein level, ELISA indicated significantly increased IL-1β and IL-6 levels in the DED group (Figure 3E,F, *p* < 0.05 vs. blank). Treatment with rlysozyme significantly reduced IL-18 mRNA at both 2.76 × 10^−5^ M and 2.76 × 10^−4^ M (Figure 3B; *p* < 0.001 vs. DED) and IL-1β at 2.76 × 10^−4^ M (Figure 3A; *p* < 0.01 vs. DED). TNF was significantly reduced at 2.76 × 10^−5^ M (Figure 3D; *p* < 0.01 vs. DED) but was not significantly different from the DED group at 2.76 × 10^−4^ M, indicating a non-monotonic dose–response for TNF. In contrast, IL-1β protein level, as well as *IL-6* mRNA and protein levels remained largely unchanged between the rlysozyme treatment group and the DED group.

To further elucidate the underlying mechanism, we examined the mRNA expression of key NLRP3 inflammasome components. Hyperosmolar stress significantly increased NLRP3 and PYD and CARD domain-containing protein (*PYCARD*) mRNA levels in the DED group (Figure 3G,H, *p* < 0.01 vs. blank), while caspase-1 mRNA showed only a modest elevation (Figure 3I). Both 0.4% hyaluronic acid (positive control) and rlysozyme treatment significantly downregulated *NLRP3* and *PYCARD* mRNA expression (*p* < 0.01 vs. DED group) in a concentration-dependent manner, whereas caspase-1 (*CASP1*) mRNA levels were not significantly affected (*N* = 3).

### 2.4. Rlysozyme Significantly Attenuates Oxidative Stress Without Altering Mitochondrial Membrane Potential

**Figure 4:**
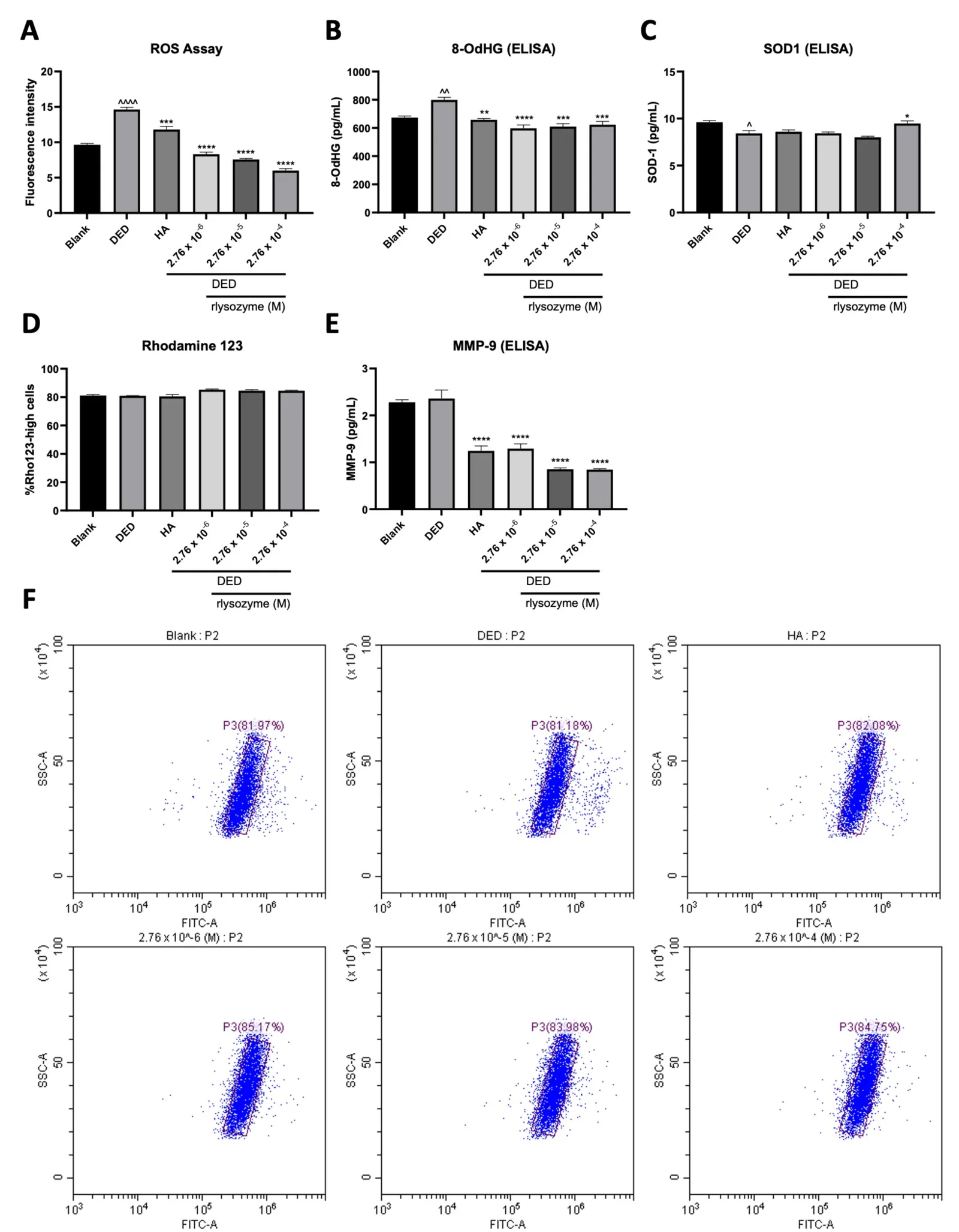
rlysozyme attenuates oxidative stress without altering mitochondrial membrane potential. (**A**) Total intracellular reactive oxygen species levels in HCEC treated with different concentrations of rlysozyme. (**B**,**C**,**E**) ELISA results showing the levels of 8-OHdG (**B**), SOD1 (**C**), and MMP-9 (**E**) in HCEC treated with various rlysozyme concentrations. (**D**) Representative Rhodamine 123 (Rho 123) scatter plots showing mitochondrial membrane potential in HCEC treated with different concentrations of rlysozyme. (**F**) Quantification of the percentage of Rho123-positive (polarized) cells in HCEC treated with different concentrations of rlysozyme. Each blue dot represents an individual cell event detected by flow cytometry. Data are presented as percentages of cells with high antioxidant capacity. Inter-group difference was determined by one-way ANOVA with Tukey’s post hoc test for multiple comparisons. *N* = 3. Statistical significance is indicated as follows: ^ *p* < 0.05, ^^ *p* < 0.01, and ^^^^ *p* < 0.0001 vs. the blank group; * *p* < 0.05, ** *p* < 0.01, *** *p* < 0.001, and **** *p* < 0.0001 vs. the DED group.

In response to hyperosmolar stress, both intracellular ROS levels and oxidative DNA damage marker 8-OHdG were significantly elevated in the DED group compared to the blank group (Figure 4A,B, *p* < 0.01 vs. blank). Treatment with rlysozyme markedly reduced ROS and 8-OHdG levels at all tested concentrations (2.76 × 10^−6^ to 2.76 × 10^−4^ M), restoring them to near blank levels (Figure 4A,B, *p* < 0.001 vs. DED group). The efficacy of rlysozyme in suppressing ROS and 8-OHdG was comparable to that of 0.4% HA. In contrast, although a slight decrease in superoxide dismutase 1 (SOD1) level was observed in the DED group (Figure 4C, *p* < 0.05 vs. blank), HA and the lower rlysozyme concentrations (2.76 × 10^−6^ to 2.76 × 10^−5^ M) did not significantly alter SOD1, whereas the highest concentration of rlysozyme (2.76 × 10^−4^ M) significantly restored SOD1 to near the blank control level (Figure 4C, *p* < 0.05 vs. DED). Similarly, *SOD2* mRNA levels were not significantly altered across groups (data not shown). Notably, total MMP-9, a downstream effector of oxidative stress, was markedly decreased by rlysozyme treatment to levels even lower than those in the blank group (Figure 4E, *p* < 0.001 vs. DED), showing comparable efficacy to that of 0.4% HA (*N* = 3). Mitochondrial membrane potential, assessed by Rho123 staining, remained stable across all groups (Figure 4D,F). The percentage of Rho123-positive cells was comparable between the DED (81.18%) and blank (81.97%) groups, indicating that hyperosmotic stress did not induce significant mitochondrial depolarization. Similarly, rlysozyme treatment (83.98–85.17%) did not significantly alter the mitochondrial membrane potential. These findings suggest that the observed antioxidative effects of rlysozyme were not accompanied by detectable changes in mitochondrial membrane potential under the tested conditions.

## 3. Discussion

DED is a complex, multifactorial immune-inflammatory disorder of the ocular surface characterized by progressive tear film destabilization [17]. Although lysozyme, a primary endogenous tear protein, is well known for its innate antimicrobial defence and anti-inflammatory properties, its direct therapeutic potential for DED has remained largely unexplored. The present study demonstrates that recombinant lysozyme treatment significantly enhances HCEC viability, accelerates wound healing, and maintains ZO-1 tight junction organization without inducing cytotoxicity. These protective effects were associated with dual modulation of oxidative stress and *NLRP3* inflammasome-related gene expression. Collectively, these findings suggest that restoring lysozyme levels may help replenish a deficient biomarker and may also attenuate the inflammatory responses, suggesting that it may warrant further investigation as a candidate therapy for DED.

The pathogenesis of DED is increasingly understood as a “vicious cycle” in which hyperosmolar stress triggers a self-perpetuating cascade of cellular damage. Central to this cycle is the interplay between oxidative stress, driven by the elevated ROS levels resulting from mitochondrial dysfunction [18], and activation of the innate immune response, including NLRP3 inflammasome assembly and pro-inflammatory cytokine release. In our DED-mimicking hyperosmolar model, HCEC exhibited increased total ROS levels and oxidative DNA damage, as confirmed by elevated 8-OHdG, a widely used indicator of guanine oxidation [19]. This is consistent with previous evidence that hyperosmotic stress induces inflammation, ROS accumulation, and oxidative stress in human corneal epithelial cells [20]. These oxidative signals, in turn, promote NLRP3 inflammasome activation, which has been implicated in multiple ocular diseases. Accordingly, NLRP3 expression was markedly upregulated approximately two-fold in DED cells relative to the blank group, consistent with transcriptional activation of the inflammasome pathway [21]. In addition, MMP-9 protein level rose markedly. MMP-9 is a well-established tear biomarker of ocular surface inflammation in DED [22]. Given its bidirectional interaction with the NLRP3 inflammasome, MMP-9 can both amplify inflammasome activity and exacerbate extracellular matrix degradation [23].

Building on these observations, recombinant lysozyme treatment significantly reduced ROS accumulation and 8-OHdG levels, suggesting reduced oxidative stress and oxidative DNA damage under the tested conditions. Total MMP-9 secretion was significantly attenuated across all treatment groups [24]. It should be noted that this ELISA-based measurement reflects total MMP-9 (pro- and active forms); assessment of the proteolytically active form of MMP-9 (e.g., by gelatin zymography) will be required in future studies to confirm functional relevance. Notably, this upstream ROS-lowering effect occurred independently of mitochondrial antioxidant pathways, as SOD1, SOD2, and Rho123 remained unchanged, despite the marked reduction in oxidative stress. In contrast, SOD1 was significantly decreased in untreated DED cells (consistent with early cytosolic antioxidant depletion), whereas stable SOD2 and Rho123 levels indicated that mitochondrial stress had not reached the threshold for adaptative response. These data suggest that rlysozyme may act via an upstream mechanism of action though a direct mechanistic link between ROS reduction and inflammasome modulation was not established in this study [25,26].

Consequently, recombinant lysozyme reduced NLRP3 expression to near-blank levels, effectively indicating downregulation of the inflammasome pathway at the transcriptional level. Although total caspase-1 protein remained unchanged, this finding is consistent with canonical NLRP3 signaling, in which NLRP3 is highly inducible under hyperosmotic stress while caspase-1 is constitutively expressed; its activation drives maturation of IL-1β and IL-18. Previous animal studies demonstrated that topical application of β-aminoarteether maleate can inhibit the NLRP3 inflammasome pathway, thereby reducing inflammation in DED animal models [27]. The lack of change therefore does not imply inactive signaling. Rather, the observed transcriptional changes are consistent with a predominant effect upstream of NLRP3-ASC complex assembly, although this was not directly assessed in the present study [28]. We only quantified total (not cleaved) caspase-1 and did not evaluate inflammasome speck formation, K+ efflux, or NIMA-related kinase 7 (NEK7) interaction, all of which may be independently modulated [29]. At the cytokine level, although rlysozyme reduced IL-1β mRNA expression, the secreted IL-1β protein level remained unchanged. This is consistent with our transcriptional-level interpretation, because IL-1β maturation and secretion require post-translational processing (caspase-1 cleavage) that was not assessed in the present study [30]. Notably, NLRP3 inflammasome selectively affected IL-1β without altering IL-6 or TNF-α. This selectivity aligns with the canonical function of NLRP3, which preferentially drives maturation of IL-1β and IL-18 rather than NF-κB-dependent cytokines such as IL-6 and TNF-α [31]. Moreover, our in vitro models lack infiltrating immune cells, which are the primary in vivo sources of IL-6 and TNF-α, resulting in a narrow dynamic range that limits detectable modulation of these cytokines [32].

By attenuating pro-inflammatory cytokines release, rlysozyme may help interrupt the secondary wave of inflammation that drives epithelial desquamation and further tear film instability. Rather than providing transient lubrication, it corrects the underlying biochemical imbalance that perpetuates DED progression. Consequently, restoring lysozyme homeostasis may help interrupt the interplay between oxidative damage and chronic inflammation, providing a robust mechanism for ocular surface recovery.

Regarding the concentrations tested, rlysozyme was applied as a 10-fold dilution series (2.76 × 10^−9^ to 2.76 × 10^−4^ M) to delineate the full dose–response relationship and identify the effective therapeutic window. The highest concentration (2.76 × 10^−4^ M, 4 mg/mL) corresponds to the maximal solubility of rlysozyme in culture medium and is only approximately two- to four-fold above the physiological lysozyme concentration in human tears (~1–2 mg/mL) [33] Since DED is characterized by reduced tear lysozyme levels [10,11,12], the doses that produced significant protective effects in this study (2.76 × 10^−5^ and 2.76 × 10^−4^ M) correspond to restoring near-physiological to modestly supraphysiological lysozyme concentrations, which are readily achievable by topical ocular instillation of a lysozyme-containing eye drop. These considerations support the translational feasibility of the selected concentrations for ocular application.

Given the chronic and multifactorial nature of DED, effective therapies must not only relieve symptoms but also promote long-term healing and safety [34]. Beyond its biochemical modulation of the inflammatory cascade, recombinant lysozyme demonstrates clear functional efficacy through physical restoration of the ocular surface. Treatment significantly accelerated corneal epithelial wound closure and preserved ZO-1 tight junction organization, key elements of the ocular surface barrier, with potency comparable to that of HA, a standard DED therapy. HA (0.4%, MW 50,000–70,000 Da) was selected as the positive control because it is a widely used lubricant [35] in clinical artificial tears at this concentration, providing a comparator for changes in tight junction organization and wound closure in the DED model [36,37]. Notably, while these data demonstrate preservation of ZO-1 structural organization, immunofluorescence alone does not establish functional barrier integrity; therefore, future studies employing transepithelial electrical resistanceor paracellular permeability assays (e.g., FITC-dextran) are warranted to definitively confirm barrier recovery. In DED, loss of these barrier proteins increases permeability and heightens susceptibility to hyperosmotic injury. By downregulating NLRP3 inflammation-related gene expression, recombinant lysozyme may foster a microenvironment conducive to cellular repair. Supporting these *in vitro* observations, preclinical evaluations of recombinant human lysozyme eye drops in a rabbit model showed improved tear secretion, corneal repair, and anti-inflammatory outcomes without irritation [38]. In addition, the cationic nature of lysozyme may enable interaction with anionic mucins in the tear film, potentially enhancing both tear film stability and surface lubrication. Thus, recombinant lysozyme serves a dual role: as a bioactive signal to quench inflammation and as a structural stabilizer to maintain ZO-1 tight junction organization. Overall, rlysozyme represents a promising multi-target alternative to existing DED treatments, such as cyclosporine A or lifitegrast, which primarily focus on late-stage T-cell mediated inflammation. By targeting the earlier vicious cycle of ROS and NLRP3 activation, it may act earlier in the disease process before irreversible ocular surface damage occurs. As an endogenous protein, recombinant lysozyme further offers superior biocompatibility and a lower risk of stinging or irritation associated with synthetic drops.

## 4. Materials and Methods

### 4.1. Materials and Reagents

Recombinant human lysozyme (rlysozyme; Cat. HKQ-201, Huikang Biotech, Xi’an, China) was used at a maximal solubility of 4 mg/mL (2.76 × 10^−4^ M) in culture medium. Dulbecco’s Modified Eagle Medium/Nutrient Mixture F-12 (DMEM/F-12, Cat. 11320033), fetal bovine serum (FBS, Cat. 10099141), penicillin–streptomycin solution (100 U/mL penicillin, 100 µg/mL streptomycin, Cat. 15140122) and Rhodamine 123 (3,6-diamino-9-(2-methoxycarbonylphenyl) ixanthylium chloride, R302) were obtained from Gibco (Thermo Fisher Scientific, Waltham, MA, USA). The MTS Cell Proliferation Assay Kit was obtained from Promega (Madison, WI, USA). Hyaluronic acid (HA, sodium hyaluronate, molecular weight 50,000–70,000 Da, 0.4%, H758878) was purchased from Aladdin (Shanghai, China). Enzyme-linked immunosorbent assay (ELISA) kits for IL-6 (Cat. EK0411), matrix metalloproteinase-9 (MMP-9, Cat. EK0465), and IL-1β (Cat. EK0392) were obtained from Lianke Biology (Hangzhou, China). ELISA kits for malondialdehyde (MDA, Cat. CEA597Ge) and 8-hydroxy-2′-deoxyguanosine (8-OHdG, Cat. CEA660Ge) were purchased from Cloud-Clone (Wuhan, China). RNeasy Plus Mini Kit (Cat. DP420, Qiagen, Valencia, CA, USA), PrimeScript RT Reagent Kit (Cat. RR037A, Takara, Japan), and TB Green^®^ Premix Ex Taq™ (Cat. RR420A, Takara, Japan) were used for RNA extraction and quantitative real-time PCR (qPCR). The ROS probe 2′,7′-dichlorodihydrofluorescein diacetate (DCFH-DA) was obtained from Beyotime Biotechnology (Cat. S0033S, Shanghai, China). For immunofluorescence, primary antibodies against zonula occludens-1 (ZO-1, Cat. 40-2200) were purchased from Invitrogen (Carlsbad, CA, USA), and Alexa Fluor^®^ 488–conjugated goat anti-rabbit IgG secondary antibodies (ab150077) were purchased from Molecular Probes (Eugene, OR, USA). Imaging was performed using a Leica TCS SPE confocal microscope (Leica Microsystems, Wetzlar, Germany).

### 4.2. Cell Culture and Treatment

Human corneal epithelial cells (HCEC; ATCC^®^ PCS-700-010™) were routinely cultured in DMEM/F-12 medium supplemented with 10% FBS and 1% Penicillin-Streptomycin. Cells were maintained at 37 °C in a humidified atmosphere containing 5% CO_2_. For experimentation, cells were seeded into 96-well plate, 24-well plate and 6-well plate for various assays. For the cytotoxicity test, cells were cultured in isosmotic medium and treated directly with lysozyme at various concentrations (2.76 × 10^−9^ to 2.76 × 10^−4^ M). For the therapeutic test, cells were exposed to hyperosmotic medium (prepared by supplementing the complete culture medium with additional sodium chloride to a final osmolarity of 450 mOsm, verified using an osmometer) for 24 h to induce the DED model when they reached a 80–90% confluency by supplementing the medium with sodium chloride to achieve a final osmolarity of 450 mOsm, as previously reported [39,40]. Upon induction, the medium was replaced with fresh treatment solutions containing recombinant human lysozyme at concentrations of 2.76 × 10^−9^ to 2.76 × 10^−4^ M (10-fold serial dilutions), 0.4% hyaluronic acid (HA, positive control) or culture medium (DED) for an additional 48 h before endpoint analyses, including MTS viability assay, wound healing, qPCR, ELISA, and immunofluorescence. A blank group was maintained in isosmotic medium (312 mOsm) throughout the experiment without any treatment.

### 4.3. Cell Viability Assay

HCEC were seeded into 96-well plates (8000 cells/well for cytotoxicity evaluation and 12,000 cells/well for efficacy evaluation) and cultured until approximately 80% confluency was reached. Safety and efficacy evaluations were conducted as described above, respectively. Upon treatment, cell viability was assessed using the MTS Cell Proliferation Assay Kit (Promega Corporation, Madison, WI, USA) following the manufacturer’s instructions. A total of 20 µL of working MTS/PMS reagent was added to each well and incubated at 37 °C for 2 h. Absorbance was measured at 490 nm using a microplate reader (BMG Labtech, Ortenberg, Germany). All results were normalized to the blank control.

### 4.4. Scratch Assay

HCEC were seeded into 24-well plates at a density of 20,000 cells/well with adjacent chambers separated by a 500 μm cell culture insert. Upon reaching 80% confluency, the insert was carefully removed to create a defined, cell-free gap (approximately 500 μm in width) between the monolayers. This gap served as the wound area for subsequent analysis. To induce DED conditions, cells were exposed to hyperosmotic medium (450 mOsm) for 24 h followed by various treatments for another 48 h as described above. Phase-contrast microscopy images were captured at 4× magnification from at least three independent fields per well at baseline (0 h) and after 48 h of treatment. The remaining wound area was quantified using ImageJ software (version 1.54f; National Institutes of Health, Bethesda, MD, USA; available at https://imagej.net/) and normalized to its initial area.

### 4.5. Intracellular ROS Levels

Intracellular ROS levels were measured using a ROS Assay Kit (Beyotime, ShanghaiChina) based on the DCFH-DA probe. After treatment, cells cultured in black 96-well plates were incubated with DCFH-DA working solution (1:250 dilution in PBS) at 37 °C for 45 min in the dark. The remaining solution was then removed, PBS was added, and fluorescence was measured at 488 nm excitation and 525 nm emission using a microplate reader.

### 4.6. RNA Extraction, Reverse Transcription, and Quantitative Real-Time PCR (RT-qPCR)

Total RNA was extracted from treated HCEC using the RNAprep Pure Micro Kit (Tiangen, China) according to the manufacturer’s instructions. RNA concentration and purity were measured using a NanoDrop ND-1000 spectrophotometer (Thermo Fisher Scientific, Wilmington, DE, USA). Complementary DNA (cDNA) was synthesized from 2.0 µg of total RNA using the PrimeScript RT Reagent Kit (Takara, Japan) according to the manufacturer’s protocol. Quantitative real-time PCR (qPCR) was performed using the QuantStudio^®^ 7 Real-Time PCR System (Applied Biosystems, Foster City, CA, USA) in a 10 µL reaction volume containing 4.4 µL of diluted cDNA, 0.2 µL of each primer pair (final concentration 0.2 µM), 0.2 µL of ROX dye and 5 µL of SYBR Green PCR Master Mix (Applied Biosystems). The thermal cycling conditions were as follows: 95 °C for 30 s, followed by 40 cycles of 95 °C for 5 s and 60 °C for 30 s. A melt curve analysis was conducted at the end of each run to confirm amplification specificity. Target genes analyzed included NLRP3, CASP1, IL-18, IL-1β, IL-6, PYCARD, MMP-9 and SOD2 with GAPDH used as the housekeeping gene. Primer sequences are listed in Table 1. All reactions were run in triplicate, and a no-template control was included to monitor for contamination. Relative gene expression was calculated using the 2^−ΔΔCt^ method.

### 4.7. Enzyme-Linked Immunosorbent Assays (ELISA)

Supernatants were collected after 48 h of treatment and centrifuged at 1000× *g* for 10 min to remove cellular debris. The concentrations of secreted IL-1β, IL-6, MMP-9, SOD1, and 8-OHdG in the supernatants were quantified using commercial ELISA kits according to the manufacturers’ instructions. Sample and standards were measured in duplicate. Absorbance for each biomarker was measured at the specified wavelength using a microplate reader (BMG Labtech, Ortenberg, Germany). Concentrations were determined from standard curves generated for each biomarker.

### 4.8. Immunofluorescence Staining

HCEC were seeded on standard glass slides at a density of 60,000 cells/slide and exposed to different treatments. Upon treatment, cells were fixed with 4% paraformaldehyde (PFA) for 15 min at room temperature. After washing with PBS, cells were blocked with 10% bovine serum albumin (BSA) in PBS for 30 min to reduce nonspecific binding. Cells were then incubated overnight at 4 °C with rabbit anti-human ZO-1 primary antibody (1:100 dilution; Invitrogen, Carlsbad, CA, USA). After washing, cells were incubated with an Alexa Fluor 488-conjugated goat anti-rabbit IgG secondary antibody (1:200 dilution; Molecular Probes, Eugene, OR, USA) for 1 h at room temperature. Nuclear staining was performed using Hoechst 33342 (Beyotime, China). Fluorescent images were captured using a Leica TCS SPE confocal microscope. ZO-1 fluorescence intensity was quantified using ImageJ (NIH, Bethesda, MD, USA). A fixed-size rectangular region of interest (ROI) was placed on each image, and the mean fluorescence intensity of the Alexa Fluor 488 (green) channel was measured.

### 4.9. Mitochondrial Membrane Potential Measurement

Mitochondrial membrane potential was measured using Rhodamine 123 (Rho123; 10 µM). After treatment, HCEC were incubated with the dye at 37 °C for 10 min in the dark, washed twice with PBS, and analyzed on a CytoFLEX flow cytometer (excitation/emission: 488/530 nm). Data were processed using CytExpert. Debris and doublets were excluded by FSC/SSC gating, and single live cells were classified based on fluorescence intensity. Rho123-high cells indicate strong mitochondrial potential and healthy bioenergetics, while Rho123-low cells reflect depolarization and dysfunction. A minor hyper-fluorescent population, associated with elevated metabolic activity or early stress adaptation, was also noted. Results were expressed as the percentage of Rho123-high cells relative to the total number of live cells.

### 4.10. Statistical Analysis

Statistical analyses were conducted using GraphPad Prism version 10 (GraphPad Software, San Diego, CA, USA). Data are presented as mean ± SEM. Inter-group differences were determined by one-way analysis of variance (ANOVA), followed by Tukey’s post hoc test for multiple pairwise comparisons. A *p* ≤ 0.05 was considered indicative of statistical significance. All experiments were conducted with a minimum of three independent biological replicates per treatment condition.

## 5. Conclusions

This study demonstrates that rlysozymeprotects human corneal epithelial cells under hyperosmotic stress, an established model of DED. Rlysozyme showed no detectable cytotoxicity even at supraphysiological concentrations under the tested conditions, indicating good in vitro biocompatibility. Functionally, it enhanced epithelial cell viability, accelerated wound closure to a level comparable to that achieved with hyaluronic acid, and preserved tight junction structural organization, while simultaneously reducing ROS accumulation and oxidative DNA damage. At the transcriptional level, rlysozyme downregulates NLRP3 inflammasome-related gene expression (*NLRP3, PYCARD*) and several pro-inflammatory cytokines (IL-1β, TNF, IL-18), whereas secreted cytokine protein levels remained largely unchanged. These changes occurred concurrently with the attenuation of the oxidative stress–inflammation axis, although their causal relationship requires further investigation. Collectively, these findings suggest that rlysozyme is a multi-target candidate for addressing oxidative stress and inflammation, supporting the rationale for further in vivo investigation of rlysozyme in DED.

## Figures and Tables

**Table 1:** Primer pairs used in RT-qPCR experiments.

Gene	Forward (5′ to 3′)	Reverse (5′ to 3′)
*PYCARD*	AGCTCACCGCTAACGTGCTGC	CCAGGCTGGTGTGAAACTGAA
*CASP1*	GCTGAGGTTGACATCACAGGCA	TGCTGTCAGAGGTCTTGTGCTC
*GAPDH*	GAGTCAACGGATTTGGTCGT	AATGAAGGGGTCATTGATGG
*IL-1β*	CCTGTCCTGCGTGTTGAAAGA	GGGAACTGGGCAGACTCAAA
*IL-6*	TGGCTGAAAAAGATGGATGCT	TCTGCACAGCTCTGGCTTGT
*IL-18*	GCTTGGCTGCCGACTGAGGAG	CCTTGATGTTATCAGGAGGATTCA
*NLRP3*	CGTGAGTCCCATTAAGATGGAGT	CCCGACAGTGGATATAGAACAGA
*SOD2*	CTGGACAAACCTCAGCCCTAAC	AACCTGAGCCTTGGACACCAAC
*TNF*	CCTGCCCCAATCCCTTTATT	CCCTAAGCCCCCAATTCTCT

## Data Availability

The raw data supporting the conclusions of this article will be made available by the authors on request.

## Abbreviations

The following abbreviations are used in this manuscript:

8-OHdG	8-Hydroxy-2′-deoxyguanosine
ANOVA	Analysis of variance
ASC	Apoptosis-associated speck-like protein containing a CARD
BSA	Bovine serum albumin
CASP1	Caspase 1
cDNA	Complementary DNA
CEVR	Centre for Eye and Vision Research
CRISPR/Cas9	Clustered regularly interspaced short palindromic repeats and CRISPR-associated protein 9
DAPI	4′,6-Diamidino-2-phenylindole
DCFH-DA	2′,7′-Dichlorodihydrofluorescein diacetate
DED	Dry eye disease
DMEM/F-12	Dulbecco’s Modified Eagle Medium/Nutrient Mixture F-12
ELISA	Enzyme-linked immunosorbent assay
FBS	Fetal bovine serum
FITC	Fluorescein isothiocyanate
FSC	Forward scatter
GAPDH	Glyceraldehyde-3-phosphate dehydrogenase
HA	Hyaluronic acid
HCEC	Human corneal epithelial cells
IgG	Immunoglobulin G
IL	Interleukin
IL-1β	Interleukin-1 beta
IL-6	Interleukin-6
IL-18	Interleukin-18
LRR	Leucine-rich repeat
LYZ	Lysozyme gene
MDA	Malondialdehyde
MMP-9	Matrix metalloproteinase-9
mRNA	Messenger RNA
MTS	3-(4,5-Dimethylthiazol-2-yl)-5-(3-carboxymethoxyphenyl)-2-(4-sulfophenyl)-2H-tetrazolium
NEK7	NIMA-related kinase 7
NF-κB	Nuclear factor kappa B
NLRP3	NOD-, LRR- and pyrin domain-containing protein 3
PBS	Phosphate-buffered saline
PFA	Paraformaldehyde
PYCARD	PYD and CARD domain-containing protein
qPCR	Quantitative polymerase chain reaction
qRT-PCR	Quantitative reverse-transcription polymerase chain reaction
Rho123	Rhodamine 123
rlysozyme	Recombinant human lysozyme
RNA	Ribonucleic acid
ROI	Region of interest
ROS	Reactive oxygen species
RT-qPCR	Reverse-transcription quantitative polymerase chain reaction
SARS-CoV-2	Severe acute respiratory syndrome coronavirus 2
SEM	Standard error of the mean
SOD1	Superoxide dismutase 1
SOD2	Superoxide dismutase 2
SSC	Side scatter
TNF	Tumor necrosis factor
TNF-α	Tumor necrosis factor alpha
ZO-1	Zonula occludens-1
